# Mercury in Ten Storm-Petrel Populations from the Antarctic to the Subtropics

**DOI:** 10.1007/s00244-023-01011-3

**Published:** 2023-07-13

**Authors:** Petra Quillfeldt, Yuliana Bedolla-Guzmán, Marcela M. Libertelli, Yves Cherel, Melanie Massaro, Paco Bustamante

**Affiliations:** 1 Department of Animal Ecology & Systematics, Justus Liebig University Giessen, Heinrich-Buff-Ring 26, 35392 Giessen, Germany; 2 Grupo de Ecología Y Conservación de Islas, A.C., Ensenada, 22800 Baja California, Mexico; 3 Departamento de Biología de los Predadores Tope, Coordinación Ciencias de la Vida, Instituto Antártico Argentino, Avenida 25 de Mayo 1143, B1650HML Buenos Aires, Argentina; 4 Centre d’Etudes Biologiques de Chizé, UMR 7372, CNRS-La Rochelle Université, 79360 Villiers-en-Bois, France; 5 School of Agricultural, Environmental and Veterinary Sciences, Gulbali Institute, Charles Sturt University, Albury, NSW 2640 Australia; 6 Littoral Environnement et Sociétés (LIENSs), UMR 7266, CNRS - La Rochelle Université, 2 Rue Olympe de Gouges, 17000 La Rochelle, France

## Abstract

The oceans become increasingly contaminated as a result of global industrial production and consumer behaviour, and this affects wildlife in areas far removed from sources of pollution. Migratory seabirds such as storm-petrels may forage in areas with different contaminant levels throughout the annual cycle and may show a carry-over of mercury from the winter quarters to the breeding sites. In this study, we compared mercury levels among seven species of storm-petrels breeding on the Antarctic South Shetlands and subantarctic Kerguelen Islands, in temperate waters of the Chatham Islands, New Zealand, and in temperate waters of the Pacific off Mexico. We tested for differences in the level of contamination associated with breeding and inter-breeding distribution and trophic position. We collected inert body feathers and metabolically active blood samples in ten colonies, reflecting long-term (feathers) and short-term (blood) exposures during different periods ranging from early non-breeding (moult) to late breeding. Feathers represent mercury accumulated over the annual cycle between two successive moults. Mercury concentrations in feathers ranged over more than an order of magnitude among species, being lowest in subantarctic Grey-backed Storm-petrels (0.5 μg g^−1^ dw) and highest in subtropical Leach’s Storm-petrels (7.6 μg g^−1^ dw, i.e. posing a moderate toxicological risk). Among Antarctic Storm-petrels, Black-bellied Storm-petrels had threefold higher values than Wilson’s Storm-petrels, and in both species, birds from the South Shetlands (Antarctica) had threefold higher values than birds from Kerguelen (subantarctic Indian Ocean). Blood represents mercury taken up over several weeks, and showed similar trends, being lowest in Grey-backed Storm-petrels from Kerguelen (0.5 μg g^−1^ dw) and highest in Leach’s Storm-petrels (3.6 μg g^−1^ dw). Among Antarctic storm-petrels, species differences in the blood samples were similar to those in feathers, but site differences were less consistent. Over the breeding season, mercury decreased in blood samples of Antarctic Wilson’s Storm-petrels, but did not change in Wilson’s Storm-petrels from Kerguelen or in Antarctic Black-bellied Storm-petrels. In summary, we found that mercury concentrations in storm-petrels varied due to the distribution of species and differences in prey choice. Depending on prey choices, Antarctic storm-petrels can have similar mercury concentrations as temperate species. The lowest contamination was observed in subantarctic species and populations. The study shows how seabirds, which accumulate dietary pollutants in their tissues in the breeding and non-breeding seasons, can be used to survey marine pollution. Storm-petrels with their wide distributions and relatively low trophic levels may be especially useful, but more detailed knowledge on their prey choice and distributions is needed.

**Supplementary Information:**

The online version contains supplementary material available at 10.1007/s00244-023-01011-3.

Marine ecosystems are major repositories of environmental contaminants. Seabirds have been widely used to monitor pollution of marine ecosystems, as they are long-lived and forage frequently at the apex of marine food webs (e.g. van Franeker and Bell 1988; Carravieri et al. 2014a, b, c). Using seabirds as bioindicators of marine contamination provides insight into the risk of human and other wildlife exposure to environmental contamination.

Mercury (Hg) is a toxic pervasive heavy metal occurring naturally in the environment. Nevertheless, anthropogenic activities, which currently represent two thirds of the global emissions (Pacyna et al. 2006) have substantially modified the cycling of this trace element on a global scale since pre-industrial times, mainly via fossil fuel combustion, industrial and agricultural residues, waste incineration and gold mining. Consequently, while Hg concentrations in the first one hundred metres in the ocean have doubled, they increased by 25% in deep waters (Lamborg et al. 2014). Hg can lead to deleterious effects on animals even at low doses, by affecting their nervous, reproductive and immune systems (Wolfe et al. 1998; Tan et al. 2009), with potential impacts at the population level (Goutte et al. 2014a, b).

High concentrations of Hg can be found not only in the vicinity of pollution sources, but also in remote environments. Because the elemental form of this metal is highly volatile and has a long atmospheric residence time (6 months to 1 year; Selin 2009), it is transported over long distances, reaching remote areas such as sub-polar and polar regions (Fitzgerald et al. 1998). When this element has been taken up from the atmosphere to the ocean, it is transformed into methyl mercury (MeHg) by microbial methylation in the water column and in sediments. Because of its high assimilation efficiency and high affinity for proteins, MeHg easily bioaccumulates in marine organisms (concentrations increase over time in their tissues) and biomagnifies in the food chains (concentrations increase at each trophic level) up to top predators resulting in elevated concentrations in seabirds. Only MeHg is biomagnified and not inorganic Hg. In seabird feathers and blood, Hg is mainly (> 90%) under the methylated form (see for instance Renedo et al. 2017, 2018), so THg is a proxy of MeHg in these tissues.

Elevated levels of MeHg contamination have been reported in seabirds from the Southern Ocean (Anderson et al. 2009; Carravieri et al. 2014a, b, c; Becker et al. 2016). In Arctic ecosystems, Dietz et al. (2009) determined that 92% of the Hg body burden accumulated in top predators result from anthropogenic activities.

As long-lived animals, seabirds accumulate dietary pollutants in their tissues in the breeding and non-breeding seasons. When the plumage is renewed, 70–90% of their accumulated Hg is excreted into their growing feathers (Agusa et al. 2005; Braune and Gaskin 1987; Honda et al. 1986). Thus, Hg concentrations in feathers reflect the year-round (long-term) Hg contamination of a bird (Thompson et al. 1998). In contrast to feathers which reflect the contamination since the last moult, blood reflects shorter-term (weeks up to 2 months) exposure (Albert et al. 2019), as well as any residual burden not yet depurated during feather moult (Bearhop et al. 2000). Thus, blood may be used to test for differences and carry-over of Hg among seasons. For example, Double-crested Cormorants (*Phalacrocorax auritus*) and Caspian Terns (*Hydroprogne caspia*) with winter sites with high Hg exposure still had elevated blood Hg values in summer (Lavoie et al. 2014).

Storm-petrels (families Oceanitidae and Hydrobatidae) are the smallest marine birds. They breed in cavities on remote, predator-free islands and most species migrate during the non-breeding season (Table 1). Their life cycle is characterized by annual breeding attempts with single-egg clutches, and a monogamous mating system (Quillfeldt et al. 2001) with intensive biparental care during incubation and chick-rearing.

**Table 1:** Summary of information available on the migration and diet of the seven storm-petrel species in this study (Winkler et al. 2020a, b)

Species	Non-breeding distribution	Breeding season diet
*Southern hemisphere species (Oceanitidae)*		
WISP	Transequatorial migrant to subtropical and temperate latitudes of N Atlantic (to 60º N off Labrador) and N Indian Ocean (to Arabian Sea), less abundant in Pacific (to 40º N; and scarce N of equator)	Dominated by crustaceans (mainly euphausiids near Antarctica, euphausiids and amphipods at subantarctic sites), also cephalopods (e.g. Ross Sea) and fish (South Georgia, South Shetlands)
BBSP	Migrates N into subtropical and tropical zones of Atlantic (to c. 10º N off W Africa and Brazil), Indian Ocean (regularly to equator) and Pacific Oceans (to c. 10º S in Coral Sea, off Solomons and S Polynesia)	Crustaceans, small fish and squid in variable amounts (e.g. South Georgia 90% crustaceans, South Shetland 50% fish, Crozet Is. 33% crustacea, 21% fish, 6% cephalopods, 39% wax and blubber particles)
GBSP	Little known, may disperse only to waters adjacent to colony (e.g. Chatham Is), regularly off SE Australia and Tasmania Only occasionally reaches subtropical waters at c. 30º N	Mainly immature barnacles (*Lepas australis*: > 80% of diet New Zealand, Kerguelen and Marion), other crustaceans (euphausiids and amphipods), also occasionally small squid and fish
WFSP	New Zealand birds move E over S Pacific to W coast of South America, not uncommon N to Galapagos	At Chatham Is (New Zealand), prey comprised 70% crustaceans and 30% fish by biomass
*Northern hemisphere species (Hydrobatidae)*		
ASSP	Year-round in California Current waters of the continental slope (200–2000 m deep) in narrow band corresponding to the shelfbreak front within 260 km from the coast	Not well known, includes small fish (myctophids), young squid and crustaceans (e.g. euphausiids, crab larvae)
BLSP	Warm waters along the coast to 39°N off northern California, south to 15°S off southern Peru, including Gulf of California, Gulf of Panama, and Gulf of Guayaquil, in shelf, shelf break, and continental slope waters	Crustaceans, small fish and squid: at San Benito (Baja California) 22–82% krill and 11–77% fish, with mainly krill in colder years and more larval fish in warmer year
LESP	Migrates to equatorial and subtropical surface waters in east Pacific, i.e. less productive waters than ASSP and BLSP	Geographically and seasonally variable amounts of small fish, crustaceans (euphausiids, decapods, amphipods, isopods, mysids, copepods), cephalopods, and jellyfish

*Species:* *ASSP* Ashy Storm-petrel, *BBSP* Black-bellied Storm-petrel, *BLSP* Black Storm-petrel, *GBSP* Grey-backed Storm-petrel, *LESP* Leach’s Storm-petrel, *WFSP* White-faced Storm-petrel, *WISP* Wilson’s Storm-petrel

In the present study, we focussed on storm-petrels from Antarctic and subantarctic breeding sites and also analysed samples from storm-petrels breeding in the North-East Pacific to enable a comparison with a less remote area. We tested for differences in the level of contamination associated with breeding and inter-breeding distribution and trophic position (determined using compound-specific stable isotope analyses). We further compared samples collected during the early and late Antarctic breeding season to quantify carry-over effects of the exposure to Hg in the inter-breeding season. Specifically, we wanted to test the hypotheses:

1. The level of contamination increases with a higher trophic position (as a result of Hg biomagnification along food webs).
2. The level of contamination increases with a more northerly breeding and inter-breeding distribution (lower contamination in Antarctic waters). For example, albatrosses feeding in Antarctic waters had lower Hg exposure compared to feeding at lower latitudes (Carravieri et al. 2014a; Cherel et al. 2018).
3. The level of contamination is higher at the beginning of the season for Antarctic species (reflecting their higher contamination with Hg at lower latitudes during the non-breeding season). We expected that over the course of the Antarctic breeding season, the contamination levels drop as birds spend more time in Antarctic waters that are less contaminated. This has been shown in black-legged kittiwakes from Svalbard, which spent their breeding season in less contaminated waters (Tartu et al. 2022), as well as in Blue petrels (Quillfeldt et al. 2022a).

## Materials and Methods

### Study Sites and Species

**Fig. 1:**
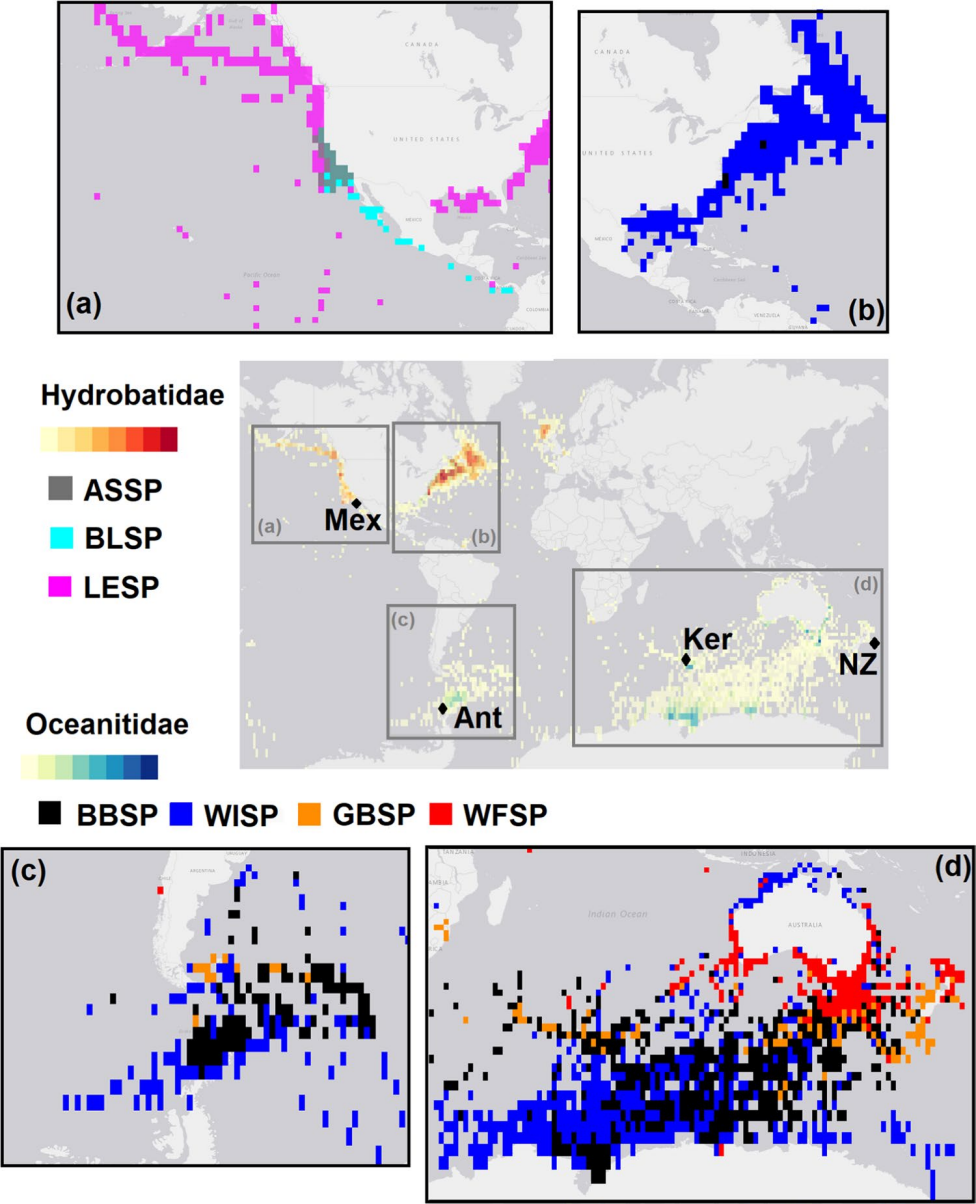
Map of the overall (multi-colony) year-round storm-petrel distributions of the seven species in this study. The central panel gives an overview of the distribution and shows the location of the study sites: Mex = Isla Coronado und Isla Todos Santos, Mexico, Ant = King George Island/25 de Mayo Island, South Shetland Islands, Antarctica, Ker = Mayes Island, Kerguelen Islands, NZ = Rangatira, Chatham Islands, New Zealand. Details are given in panel **a**–**d**: **a** Northern hemisphere species, **b** Northern hemisphere distribution of non-breeding Antarctic Storm-petrels. **c** + **d** Southern hemisphere species. Species: ASSP = Ashy Storm-petrel, BBSP = Black-bellied Storm-petrel, BLSP = Black Storm-petrel, GBSP = Grey-backed Storm-petrel, LESP = Leach’s Storm-petrel, WFSP = White-faced Storm-petrel, WISP = Wilson’s Storm-petrel, Distribution data and map source: https://mapper.obis.org

Adult birds were generally captured by hand or mistnet in the colony when arriving from foraging after nightfall. All birds were released at the location of capture immediately after sampling. The present knowledge on the distribution and diet (Fig. 1) of the species (Winkler et al. 2020a, b) is summarized in Table 1.

In the Antarctic, we sampled Wilson’s Storm-petrels (WISP) *Oceanites oceanicus* and Black-bellied Storm-petrels (BBSP) *Fregetta tropica* breeding at the Tres Hermanos (Three Brothers Hill) colony on King George Island/25 de Mayo Island, South Shetland Islands (62°14’S, 58°40’W). Birds were trapped using a 12 m mistnet between 20 November and 26 December 2017 (incubation), and 30 January and 28 February 2020 (chick-rearing). WISP and BBSP lay eggs from mid-December, and the peak of chick hatching is in the first half of February, but there is considerable variation in hatching date within the colony (e.g. 49 d time span in 1996; Quillfeldt and Peter 2000). Chicks are fed by their parents until fledging in the second half of March. The two sympatric species differ in their diet composition during the breeding season. While Black-bellied Storm-petrels take fish and crustaceans in equal proportions (Hahn 1998), Wilson’s Storm-petrels take mainly crustaceans (80–90% occurrence), feeding predominantly on Antarctic krill *Euphausia superba* (Quillfeldt 2002). Preliminary results from these two populations were presented in a conference (Quillfeldt et al. 2022b).

In the subantarctic, we sampled WISP and BBSP, as well as Grey-backed Storm-petrels (GBSP) *Garrodia nereis* breeding at Ile Mayes (49°28’S, 69°57’E) in the Kerguelen archipelago. Birds were trapped using mistnets between 5 and 12 December 2018 (incubation), and 22 February and 9 March 2019 (chick-rearing). In Crozet Islands in the Indian Ocean, WISP took mainly amphipods *Themisto gaudichaudii* and *Euphausia vallentini*, together with some copepods and cyprid larvae of cirripeds (Ridoux 1994). BBSP took the same crustaceans, but most of their prey was made up of fish and squid most likely obtained by scavenging (Ridoux 1994). GBSP have a diet specialized on Cirripedia (barnacle) larvae such as those of *Lepas australis*, a cold-water species inhabiting all the oceans surrounding the Antarctic continent (Newman an Ross 1971; Hinojosa et al. 2006). These pelagic barnacles attach to flotsam such as kelp and debris after spending up to 2 months drifting as larvae (Hinojosa et al. 2006). They are omnivores feeding on crustaceans and diatoms, and are among the higher trophic level animals in drifting seaweed communities (Sano et al. 2003).

In New Zealand, fieldwork was carried out in 2015 on South East Island (Hokorereoro/Rangatira: 44° 20′ S, 176° 10′ W). We collected samples from GBSP and White-faced Storm-petrels (WFSP) *Pelagodroma marina* from 25 November to 6 December 2015 (late incubation/hatching period). These data have previously been compared to other sympatric petrel species (Thébault et al. 2021).

In the Pacific Ocean, we sampled Black Storm-petrels (BLSP) *Hydrobates melania*, Leach’s Storm-petrels (LESP) *Hydrobates leucorhous* and Ashy Storm-petrels (ASSP) *Hydrobates homochroa* on Coronado Island (32°27′ N, 117°18′ W) from 11 to 15 July 2018 and additionally, Ashy Storm-petrels at Todos Santos Sur Island (31°48′ N, 116°47′ W) from 16 to 18 July 2018 (during the chick-rearing season).

### Sample Preparation

*Body feathers* were plucked and conserved in sealed plastic bags until analysis. Body feathers are commonly considered as the best feather type to collect, since they are more representative of the entire plumage than other feather types and more homogenous (Furness et al. 1986). From a subsample of birds (*N* = 6 per colony), we analysed 4–12 individual feathers to compare the variability in stable isotope and Hg values within and among birds (361 feathers from 51 birds). Based on the results we obtained, the samples of the remaining birds were pooled for each individual for analysis.

Feathers were cleaned in a chloroform: methanol solution (2:1, *v/v*) in an ultrasonic bath and rinsed twice in methanol. After 48 h of drying at 45 °C in an oven, feathers were cut into tiny fragments with stainless steel scissors in order to obtain a homogenous powder.

*Blood* (0.2–0.4 ml) was sampled by puncturing the brachial wing vein and collected using heparinized capillaries, or using 0.3 ml syringes. Blood was stored and transported in ethanol. Storage in ethanol has been generally found to not affect the stable isotope values of blood samples (e.g. Hobson et al. 1997; Halley et al. 2008, but see Bugoni et al. 2008). In the laboratory blood samples were freeze-dried and ground to powder to be analysed for Hg and stable isotopes.

The half-life of isotope turnover for avian red blood cells in crows *Corvus brachyrhynchos* was 29.8 d (Hobson and Clark 1993) and 10.9 d for Yellow-rumped Warblers *Dendroica coronate* (Podlesak et al. 2005). Thus, blood samples collected from the small petrels in the present study likely represented the diet ingested ca. 2–4 weeks before sampling. Upon return to their breeding grounds, stable isotope values switch to values characteristic of their breeding season diet and location, suggesting minimal carry-over of isotopic signatures from diet and foraging areas during the non-breeding season (Lavoie et al. 2014).

However, significant carryover of Hg among seasons and slow changes in Hg over time have been observed, especially in individuals with high Hg exposure during non-breeding months (Lavoie et al. 2014; Quillfeldt et al. 2022a). This suggests that Hg is stored for long time periods in internal tissues and has a slow depuration rate. Hg is retained in the organism because renal excretion of MeHg is low and bile excretion is followed by intestinal reabsorption (Norseth and Clarkson 1971; Hirata and Takahashi 1981). A Hg carryover from wintering (non-breeding) sites indicates that Hg values measured in blood at a given time may be influenced by current uptake as well as the previous exposure from distant locations.

### Mercury Analyses

Hg concentrations were determined as described in Bustamante et al. (2006). We measured aliquots (blood ~ 2 mg dry weight (dw), feathers ~ 1 mg dw) with an Advanced Mercury Analyser spectrophotometer Altec AMA-254. Measurements were repeated 2–3 × for each sample, until the relative standard deviation (RSD) was < 10%. For each measurement, accuracy and reproducibility were tested by including analytical blanks and certified reference materials (TORT-2: lobster hepatopancreas, certified concentration: 0.27 ± 0.06 μg g^−1^ dw; DOLT-5: dogfish liver, certified concentration: 0.44 ± 0.18 μg g^−1^ dw; National Research Council of Canada). Hg concentrations measured for the reference materials were: 0.26 ± 0.02 μg g^−1^ dw (*n* = 18) and 0.44 ± 0.01 μg g^−1^ dw (*n* = 7) for TORT-2 and DOLT-5 corresponding to a recovery rate of 96 ± 7% for TORT-2 and 100 ± 2% for DOLT-5. The limit of detection was 0.005 μg g^−1^ dw. Hg concentrations are expressed in μg g^−1^ dw.

### Bulk Stable Isotope Analyses

In the Southern Ocean, δ^13^C values of seabirds correspond to the latitude of their foraging habitats (Quillfeldt et al. 2010; Jaeger et al. 2010), while δ^15^N values increase with trophic level (Cherel et al. 2010).

For bulk stable isotope analyses, an amount of 0.2–0.4 mg of subsample was weighed into tin cups. Carbon and nitrogen isotopic values were measured with a continuous-flow mass spectrometer (Thermo Scientific Delta V Advantage) coupled to an elemental analyser (Thermo Scientific Flash EA 1112). Internal laboratory standards (acetanilide and peptone) indicated a precision of ± 0.15‰ for both elements. Results are expressed in parts per thousand (‰) in the δ notation, relative to Vienna Pee Dee Belemnite for δ^13^C and atmospheric N_2_ for δ^15^N, following the formula:

$$\delta^{13} {\text{C or }}\delta^{15} {\text{N}} = \left( {\frac{{R_{{{\text{sample}}}} }}{{R_{{{\text{standard}}}} }} - 1} \right) \times 10^{3}$$

where *R* is ^13^C/^12^C or ^15^N/^14^N, respectively.

In the Southern Ocean, we followed Cherel et al. (2018) to define distribution zones, based on feather δ^13^C isoscapes (Jaeger et al. 2010), as follows: Subtropical Zone (STZ): δ^13^C >  − 18.3 ‰, Subantarctic Zone (SAZ): δ^13^C values of, − 21.2 to − 18.3 ‰, and Antarctic Zone (AZ): δ^13^C <  − 21.2 ‰.

### Compound-Specific Isotope Analyses of Amino Acids (CSIA-AA)

CSIA-AA can be used to provide a baseline-independent estimate of the trophic position of marine organisms from temporally and spatially variable environments. Therefore, it is especially suitable for this dataset spanning samples from different ocean basins and latitudes and thus, with different bulk stable isotope baselines.

CSIA-AA were performed at the UC Davis Stable Isotope facility (USA), as described previously (Quillfeldt & Masello 2020). Trophic positions (TP) were calculated from nitrogen stable isotope values of glutamic acid (Glx) and phenylalanine (Phe), using a stepwise trophic discrimination factor (multi-TDF_Glx-Phe_, with the equations:

$${\text{TP}}\left[ {{\text{feathers}}} \right] = 2 + \frac{{{\text{Glx}} - {\text{Phe}} - 3.5 \permille - 3.4 \permille}}{6.2 \permille}$$

$${\text{TP}}\left[ {\text{blood cells}} \right] = 2 + \frac{{{\text{Glx}} - {\text{Phe}} - 4 \permille - 3.4\permille}}{6.2 \permille}$$

For a detailed discussion, see Quillfeldt and Masello (2020). Small sample sizes were analysed with CSIA-AA due to the high costs (Tables 2 and 3).

**Table 2:** Feather Hg concentrations and stable isotope values (mean ± SD) of Storm-petrels from ten populations

Species	Site	*n* (Hg)	THg (μg g^−1^ dw)	Range (mean)	Range (ind)	*n* (SIA)	δ^13^C (‰)	δ^15^N (‰)	*n* (CSIA)	TP (CSIA)
*Southern hemisphere species (Oceanitidae)*										
WISP	KGI	60	2.22 ± 0.94	0.93–3.12	0.65–4.57	50	−18.5 ± 0.9	13.9 ± 0.6	5	3.36 ± 0.29
	KER	12	0.64 ± 0.24	0.22–1.05	0.18–1.80	31	−17.2 ± 0.4	15.0 ± 0.5	5	3.24 ± 0.09
BBSP	KGI	44	6.19 ± 2.23	3.38–10.48	3.06–12.45	40	−17.4 ± 0.8	13.8 ± 0.6	5	3.83 ± 0.11
	KER	2	2.94 ± 0.51	2.42–3.45	1.81–5.49	2	−17.7 ± 0.1	13.5 ± 0.9	2	3.82 ± 0.14
GBSP	KER	7	0.48 ± 0.15	0.26–0.66	0.19–1.14	8	−18.0 ± 0.3	10.0 ± 0.2	4	3.61 ± 0.04
	CHA	10	0.49 ± 0.22	0.13–0.99	NA	10	−17.7 ± 0.2	9.5 ± 0.3	5	3.82 ± 0.11
WFSP	CHA	10	1.66 ± 0.48	1.05–2.61	NA	10	−16.6 ± 0.2	12.0 ± 1.5	5	3.52 ± 0.40
*Northern hemisphere species (Hydrobatidae)*										
ASSP	MEX	20	7.04 ± 1.99	3.50–12.73	2.34–15.13	20	−18.0 ± 0.3	17.9 ± 1.1	–	–
BLSP	MEX	31	5.58 ± 2.38	3.97–8.82	1.42–14.23	31	−16.4 ± 0.3	15.6 ± 0.4	5	3.44 ± 0.24
LESP	MEX	10	7.58 ± 2.35	3.44–11.37	3.25–25.01	10	−17.4 ± 0.3	15.0 ± 0.5	–	–
Kruskal–Wallis ANOVA			χ^2^ = 162.4, *df* = 9, *P* < 0.001				χ^2^ = 127.8, *df* = 9, *P* < 0.001	χ^2^ = 158.5, *df* = 9, *P* < 0.001		χ^2^ = 21.8, *df* = 7, *P* = 0.003

*Species:* *ASSP* Ashy Storm-petrel, *BBSP* Black-bellied Storm-petrel, *BLSP* Black Storm-petrel, *GBSP* Grey-backed Storm-petrel, *LESP* Leach’s Storm-petrel, *WFSP* White-faced Storm-petrel, *WISP* Wi*lson’s Storm-petrel. Site abbreviations: KGI King George Island/25 de Mayo Island (South Shetlands, Antarctic), KER Kerguelen (Southern Indian Ocean), CHA* Chatham Islands, New Zealand, *MEX* Mexican Pacific Islands. Range (mean) gives the range for mean values for individual birds, and Range (ind) for individual feathers

The sample size refers to individuals. *NA* not analysed

**Table 3:** Blood Hg concentrations and stable isotope values

Species	Site (period)	*n* (Hg)	THg (μg g^−1^ dw)	*n* (SIA)	δ^13^C (‰)	δ^15^N (‰)	*n* (CSIA)	TP (CSIA)
*Southern hemisphere species (Oceanitidae)*								
WISP	KGI (inc.)	33	0.89 ± 0.37	33	−21.9 ± 1.1	10.9 ± 0.7	5	3.44 ± 0.13
	KGI (late)	16	0.50 ± 0.24	17	−25.9 ± 0.5	9.2 ± 0.4		
	KER (inc.)	12	0.70 ± 0.21	12	−20.2 ± 0.3	10.6 ± 0.5	5	3.30 ± 0.13
	KER (late)	19	0.68 ± 0.47	19	−20.9 ± 0.8	9.7 ± 0.5		
BBSP	KGI (inc.)	21	2.46 ± 0.42	21	−23.7 ± 0.7	10.7 ± 0.3	5	3.84 ± 0.06
	KGI (late)	18	2.76 ± 0.73	19	−25.4 ± 0.4	10.1 ± 0.5		
	KER (late)	2	2.05 ± 0.31	2	−22.0 ± 0.1	9.9 ± 0.3	2	3.59 ± 0.12
GBSP	KER (inc.)	6	0.16 ± 0.03	6	−19.3 ± 0.1	9.2 ± 0.1	4	3.36 ± 0.09
	KER (late)	2	0.08 ± 0.02	2	−19.3 ± 0.1	9.2 ± 0.1		
	CHA (late)	10	0.56 ± 0.15	10	−19.0 ± 0.1	9.8 ± 0.4	5	3.54 ± 0.12
WFSP	CHA (late)	10	0.92 ± 0.24	10	−19.1 ± 0.2	9.8 ± 0.4	5	–
*Northern hemisphere species (Hydrobatidae)*								
ASSP	MEX (late)	20	1.93 ± 0.82	20	−19.4 ± 0.3	15.9 ± 0.4	–	–
BLSP	MEX (late)	31	2.60 ± 0.70	31	−19.0 ± 0.2	15.5 ± 0.3	5	3.62 ± 0.38
LESP	MEX (late)	10	3.55 ± 0.55	10	−19.7 ± 0.5	15.4 ± 0.3	–	–
Kruskal–Wallis ANOVA			χ^2^ = 166.9, *df* = 9, *P* < 0.001		χ^2^ = 180.0, *df* = 9, *P* < 0.001	χ^2^ = 145.6, *df* = 9, *P* < 0.001		χ^2^ = 24.9, *df* = 6, *P* < 0.001

*Species*: *ASSP* Ashy Storm-petrel, *BBSP* Black-bellied Storm-petrel, *BLSP* Black Storm-petrel, *GBSP* Grey-backed Storm-petrel, *LESP* leach’s Storm-petrel, *WFSP* White-faced Storm-petrel, *WISP* Wilson’s Storm-petrel. Site abbreviations: *KGI* King George Island/25 de Mayo Island (South Shetlands, Antarctic), *KER* Kerguelen (Southern Indian Ocean), *CHA* Chatham Islands, New Zealand, *MEX* Mexican Pacific Islands. Kruskal–Wallis ANOVA tests correspond to comparisons among colonies (incubation and chick-rearing combined)

### Distribution Data

**Fig. 2:**
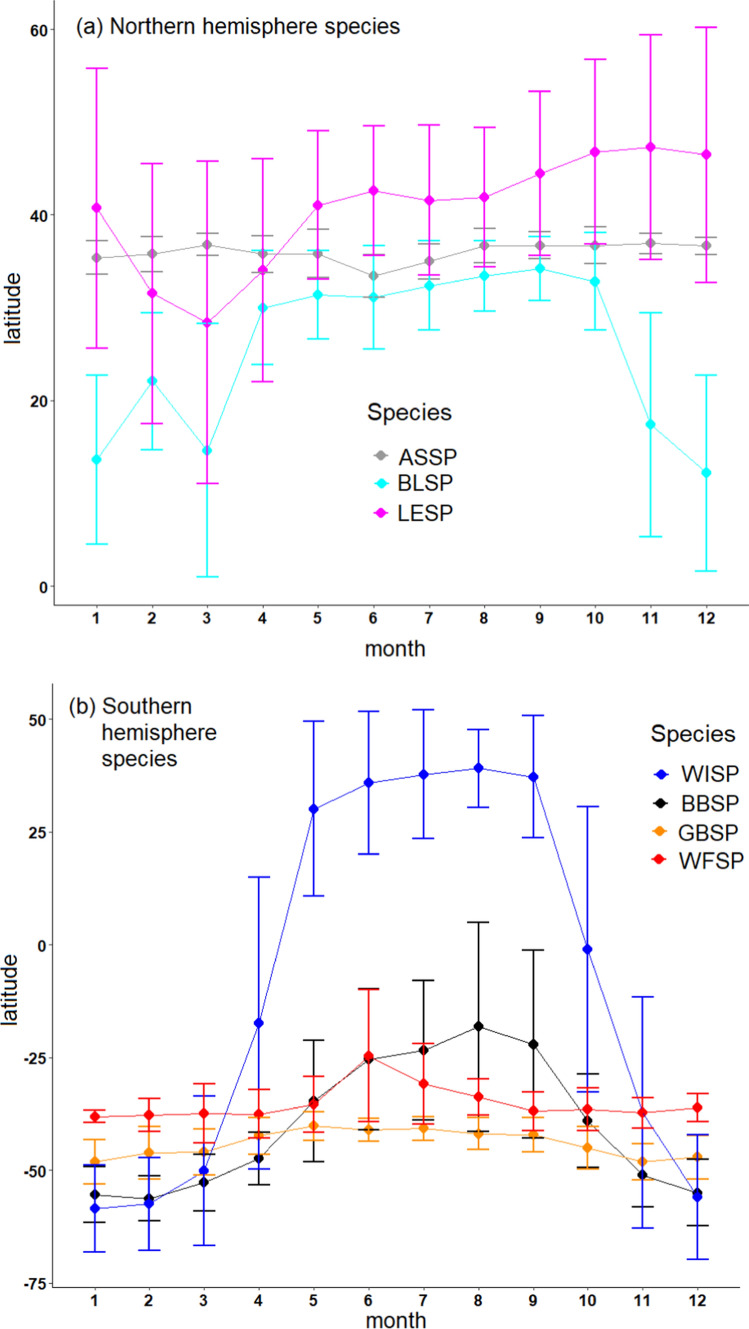
Overall (multi-colony) year-round latitudinal distribution (mean and standard deviation) of storm-petrels in this study. Distribution data were downloaded from gbif.org. Species: ASSP = Ashy Storm-petrel, BBSP = Black-bellied Storm-petrel, BLSP = Black Storm-petrel, GBSP = Grey-backed Storm-petrel, LESP = Leach’s Storm-petrel, WFSP = White-faced Storm-petrel, WISP = Wilson’s Storm-petrel

Distribution data were downloaded from GBIF.org on 08 October 2021, and plots for Fig. 1 from OBIS-SEAMAP (https://mapper.obis.org/, Halpin et al. 2009). GBIF data were summarized monthly (Fig. 2) and seasonally: the winter season was defined as June to August for southern hemisphere species, and December to February for northern hemisphere species. For WFSP, only southern hemisphere records were counted to exclude the northern hemisphere populations of this species.

### Data Analyses

Data were analysed and visualized in R version 4.1.0. We used Shapiro tests and qq plots to test normality. Stable isotope and Hg values differed significantly from normal distribution. Therefore, univariate statistics on these were carried out with nonparametric tests, and the data were transformed using transform Tukey in the R package rcompanion before carrying out multivariate statistics such as linear models. A comparison of the model outputs did not show any major differences between models using transformed and untransformed data. To enhance readability, effect plots are thus given from models of untransformed data, i.e. showing the actual scale of the data. Means are given with standard deviations. To measure effect size, we included eta squared values (*η*^2^), obtained with the EtaSq function in the R package DescTools. To quantify intra-individual variabilities in stable isotope vales, we calculated repeatability values from six birds per species (but only two Black-bellied Storm-petrels from Kerguelen) following Lessels and Boag (1987).

To test nonlinear effects, Hg values were modelled using General additive models (GAMs) in the ‘mgcv’ package in R (Wood 2021). Cross-species models were developed separately for the northern and southern hemisphere populations, including species as random factor. In addition, separate models were developed for the populations. Models included δ^13^C and δ^15^N as fixed factors. The smoothing parameter was chosen automatically using generalized cross-validation. Contour plots were generated with the vis.gam function.

## Results

### Distribution

The distribution data of the three northern hemisphere species from sightings corresponding to multiple colonies (Fig. 2, upper panel) showed that ASSP remain close to their breeding sites in the winter (November–March), while BLSP move to more southern (i.e. tropical) waters. The mean distribution of Black Storm-petrels was in the tropics (< 23.3°S) between November and March. Leach’s Storm-petrels LESP had a wide distribution that overlapped with the latitude of their breeding distribution throughout the year. Overall, LESP tended to be more northerly in early winter (November–January) and in more southern latitudes in late winter (February–March).

Among the southern hemisphere species (Fig. 2, lower panel), GBSP remained close to the latitude of their breeding colony in the winter (April to October). WISP migrated to the northern hemisphere (mean lat. 37 °N) and BBSP and WFSP moved to subtropical and tropical waters. The mean distribution of BBSP was in the tropics (≥ − 23.3°S) in August and September.

### Feather Samples

**Fig. 3:**
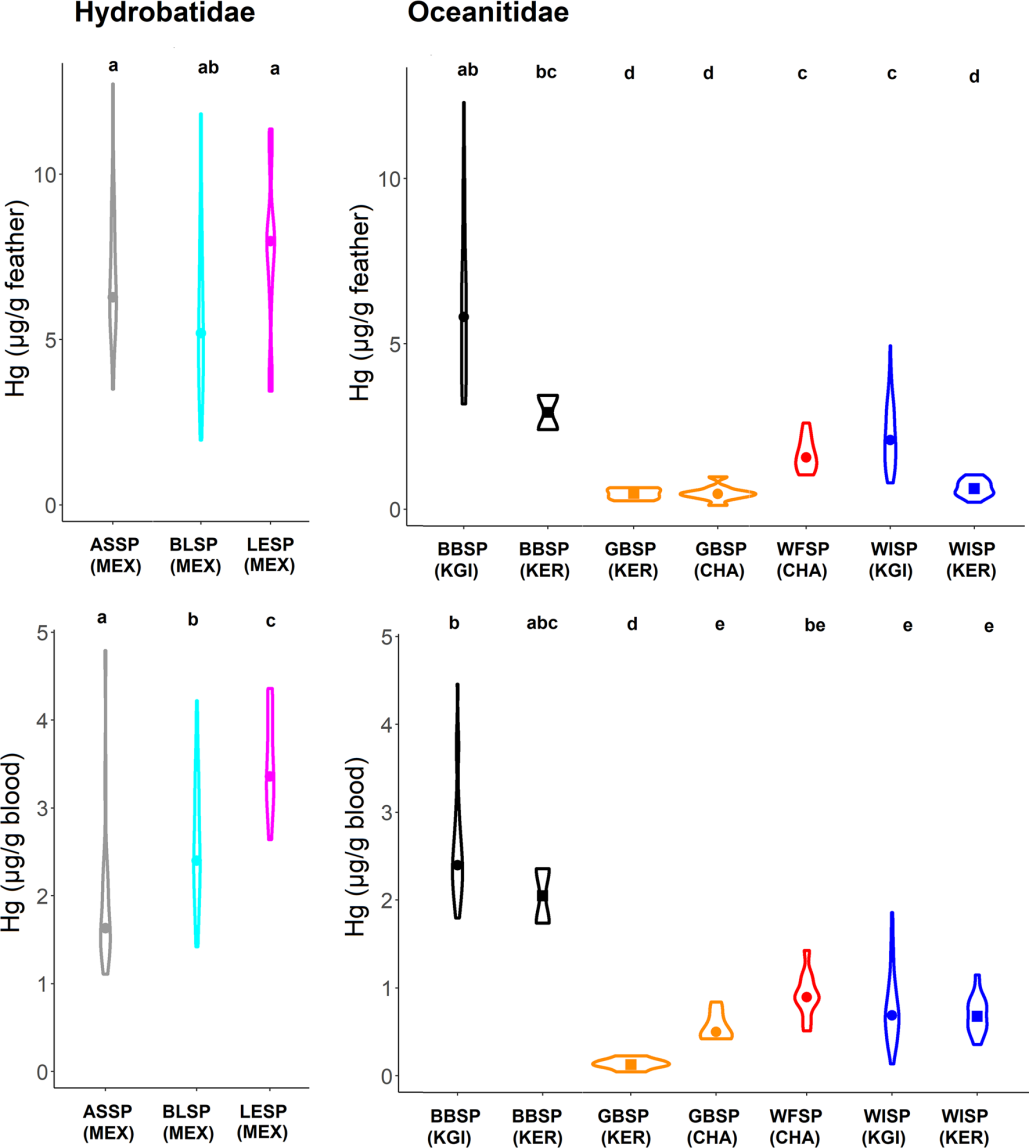
Hg concentrations in feathers and blood samples of storm-petrels from ten populations. Species: ASSP = Ashy Storm-petrel, BBSP = Black-bellied Storm-petrel, BLSP = Black Storm-petrel, GBSP = Grey-backed Storm-petrel, LESP = Leach’s Storm-petrel, WFSP = White-faced Storm-petrel, WISP = Wilson’s Storm-petrel, Sites: KGI = King George Island/25 de Mayo Island (South Shetlands, Antarctic), KER = Kerguelen Islands (Southern Indian Ocean), MEX = Mexican Pacific Islands, CHA = Chatham Islands, New Zealand

Low Hg concentrations < 5 μg g^−1^ were found in feathers of southern hemisphere species (Table 2, Fig. 3), with the exception of BBSP from King George Island/25 de Mayo Island. The Hg concentrations of those storm-petrels were close to those of the three northern hemisphere species, with elevated Hg concentrations > 5 μg g^−1^ (Table 2, Fig. 3). Among Antarctic storm-petrels, BBSP had threefold higher values than WISP. In both species, birds from the South Shetlands (Antarctica) had threefold higher values than birds from Kerguelen (Subantarctic Indian Ocean; *t*-test for BBSP: *t* = 5.3, *df* = 2.1, *P* = 0.032, *t*-test for WISP: *t* = 11.2, *df* = 65.4, *P* < 0.001).

**Fig. 4:**
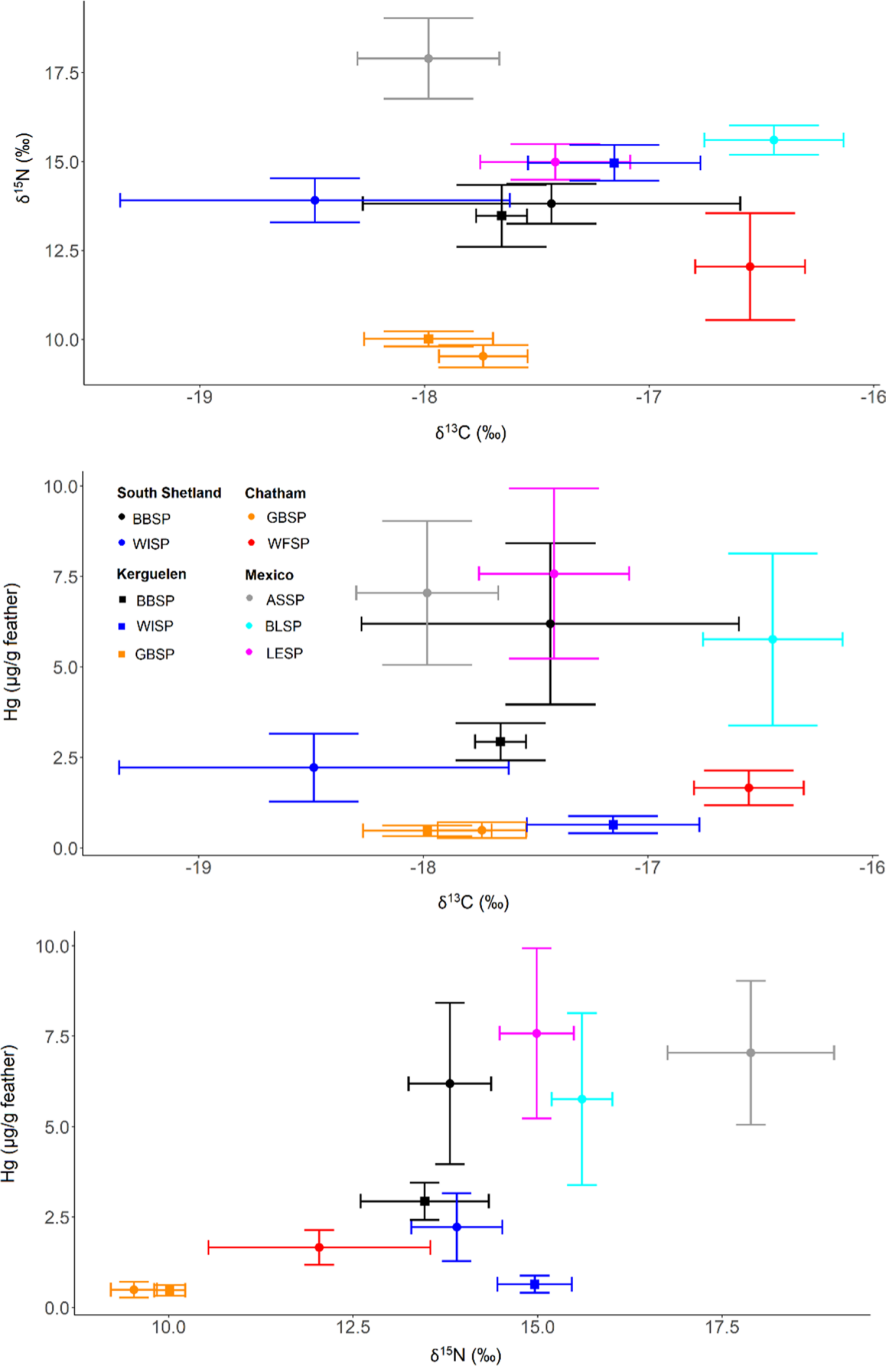
Hg concentrations and stable isotope values in feathers of storm-petrels from ten populations. Species: ASSP = Ashy Storm-petrel, BBSP = Black-bellied Storm-petrel, BLSP = Black Storm-petrel, GBSP = Grey-backed Storm-petrel, LESP = leach’s Storm-petrel, WFSP = White-faced Storm-petrel, WISP = Wilson’s Storm-petrel

The carbon stable isotope values of feathers showed a relatively narrow range (SD within − 19.5 to − 16.0 ‰, Fig. 4), indicating a wintering range from subtropical to temperate waters. Nitrogen stable isotope values had a larger range (9–19 ‰, Fig. 4). In a linear model across species, carbon stable isotope values did not correlate with Hg concentrations (*F* = 0.2, *P* = 0.653), but nitrogen stable isotope values were positively related to Hg concentrations (*F* = 7.9, *P* = 0.026, Fig. 4). Similarly, in models run across species, the trophic position according to CSIA-AA had a positive correlation with feather Hg values, while carbon stable isotope values did not correlate with Hg concentrations (Fig. S1). Overall, however, the strongest effects were those of species and site differences (Fig. S1).

The trophic position according to CSIA-AA measured in feathers ranged from 2.9 to 4.0 (Table 2, Fig. S2), while the ranges for the species overlapped, a 0.5 TP difference was seen within the Antarctic storm-petrels, with higher trophic levels in BBSP than WISP (Fig. S2).

Feathers analysed individually for repeatability of Hg concentrations (*N* = 6 individuals per colony, 4–12 individual feathers per bird) showed different degrees of repeatability, from *r* < 0 (MEX_BLSP), over poor repeatability < 50% (MEX_LESP: 18%, KER_BBSP: 40%, KER_GBSP: 48%, KER_WISP: 48%), to moderate repeatability > 50% (KGI_WISP: 52%, MEX_ASSP: 58%). Only BBSP from King George Island/25 de Mayo Island (Antarctica) reached good repeatability > 80% (KGI_BBSP: 81%, Fig. S3).

### Blood Samples

Species differences in Hg in blood samples mirrored those in feather samples, but at a narrower range (Fig. 3). Low Hg concentrations < 1.5 μg g^−1^ dw were found in feathers of the southern hemisphere species (Table 3, Fig. 3), with the exception of the BBSP. The Hg concentrations of those BBSP were close to those of the three northern hemisphere species, with elevated Hg concentrations > 5 μg g^−1^ (Table 2, Fig. 3). Among Antarctic storm-petrels, BBSP had threefold higher values than WISP. In contrast to Hg concentrations in feathers, birds from the South Shetlands (Antarctica) had similar blood Hg values to birds from Kerguelen (Subantarctic Indian Ocean; *t*-test for BBPS: *t* = 1.7, df = 1.3, *P* = 0.308, *t*-test for WISP: *t* = 0.8, *df* = 62.5, *P* = 0.385).

**Fig. 5:**
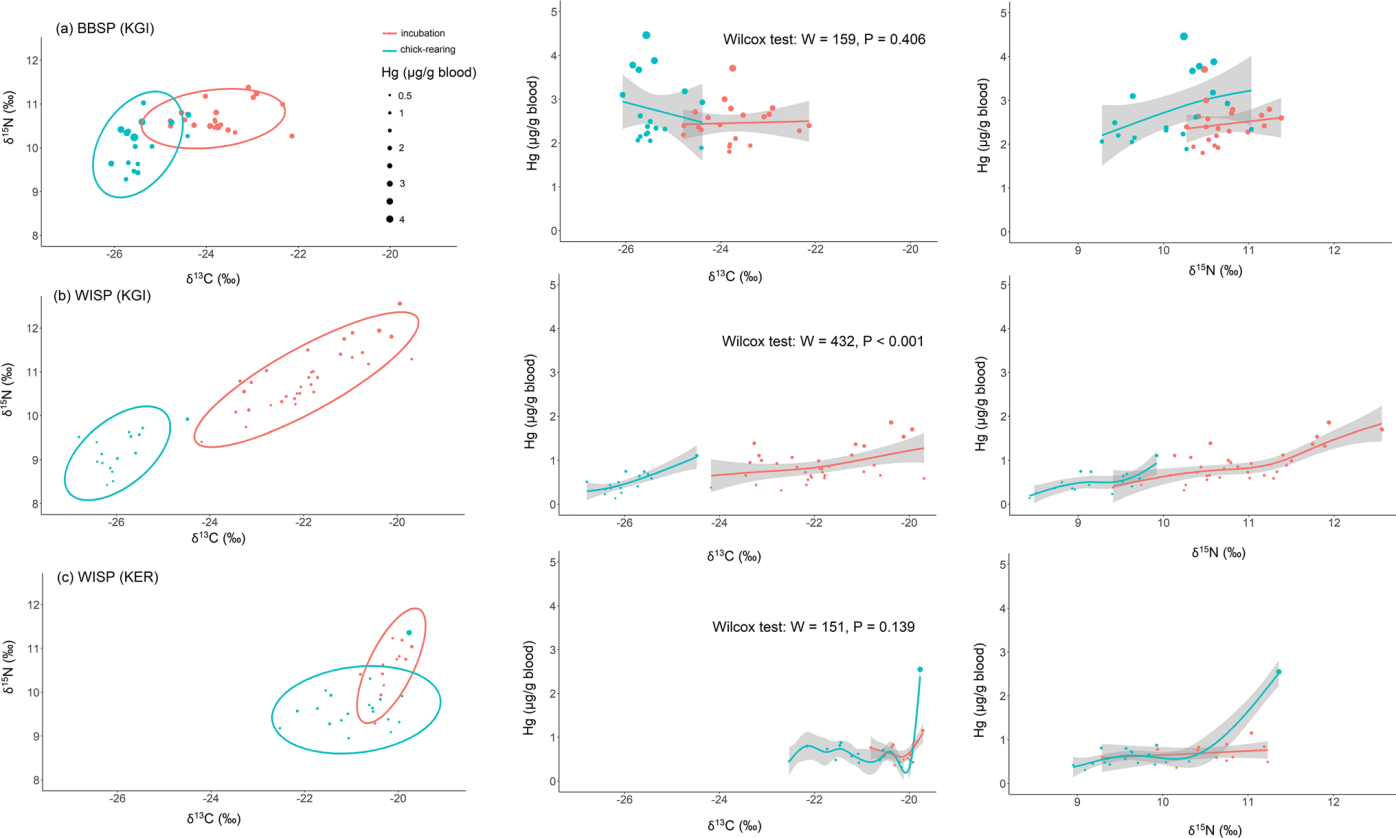
Temporal changes in Hg concentrations in blood of Wilson’s and Black-bellied Storm-petrels over the breeding season, in relation to stable isotope values. The size of the points is relative to the Hg concentration, and Wilcoxon tests correspond to comparisons of Hg concentrations between incubating and chick-rearing birds. Species: BBSP = Black-bellied Storm-petrel, WISP = Wilson’s Storm-petrel, Sites: KGI = King George Island/25 de Mayo Island (South Shetlands, Antarctic), KER = Kerguelen Islands (Southern Indian Ocean)

In Antarctic storm-petrels, Hg concentrations in blood consistently increased with increasing δ^15^N values (Figs. 5 and S4). We used GAMs to test for effects of foraging habitat (δ^13^C), trophic position (δ^15^N) and breeding stage (incubation vs. chick-rearing) on Hg concentrations in blood of Antarctic storm-petrels. We found increasing blood Hg with higher δ^15^N (Figs. S4 and 6, Table 4), while the influence of δ^13^C was both weaker (lower effect sizes), and less consistent in direction (Fig. S4). The breeding stage played a minor role, although Hg concentrations decreased in WISP on King George Island/25 de Mayo Island over the course of the breeding season (Fig. S4).

**Fig. 6:**
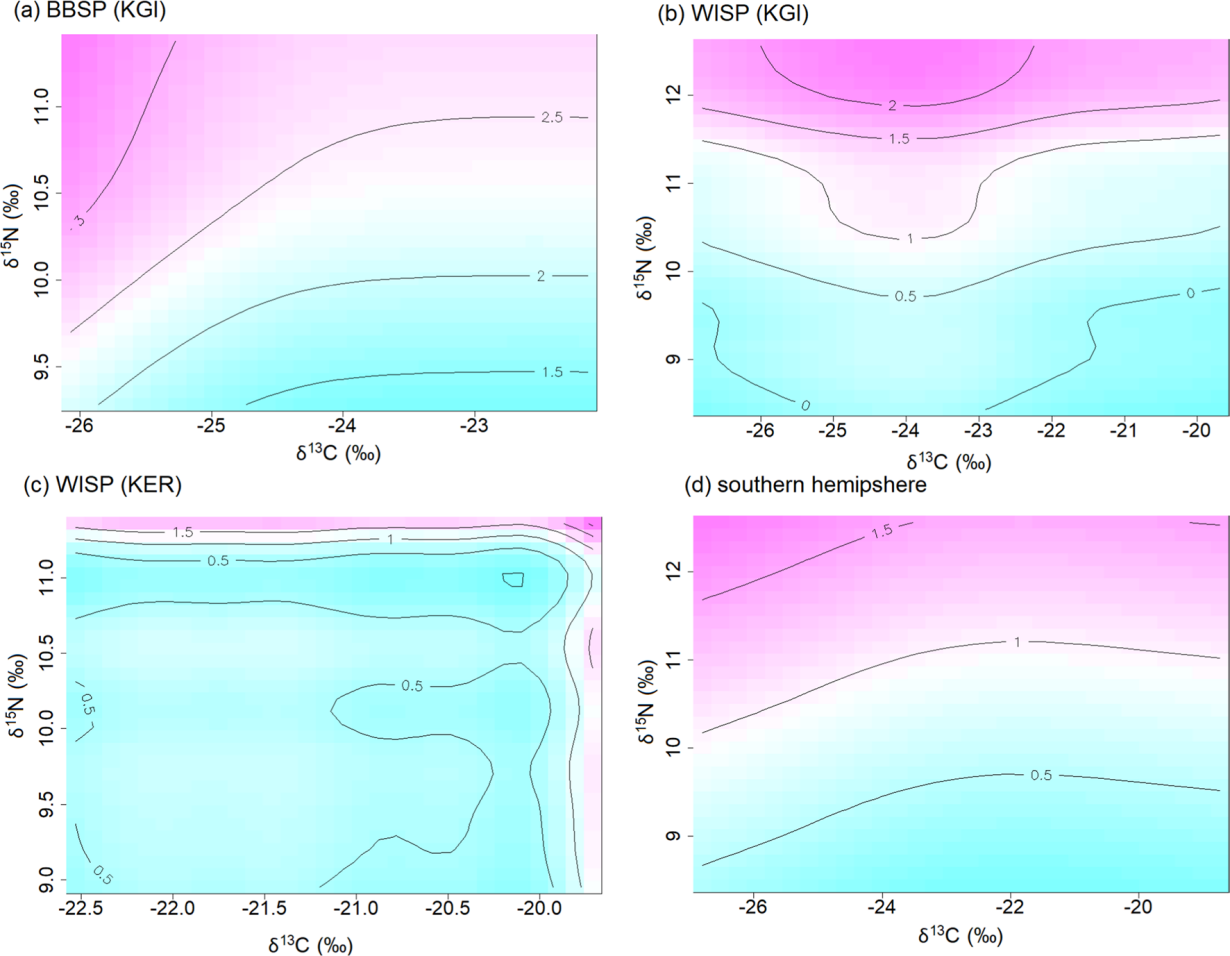
General additive model (GAM) fits of Hg values in feathers of storm-petrels breeding on King George Island/25 de Mayo Island (South Shetland, Antarctic) and Kerguelen Islands, in relation to carbon and nitrogen stable isotope values and the time in the breeding season. Species: BBSP = Black-bellied Storm-petrel, WISP = Wilson’s Storm-petrel, Sites: KGI = King George Island/25 de Mayo Island (South Shetlands, Antarctic), KER = Kerguelen (Southern Indian Ocean)

**Table 4:** Generalized Additive Model (GAM) results for blood Hg values of storm-petrels as a function of distribution (δ^13^C), trophic position (δ^15^N) and species

Species_Site	Variable	Smoother edf (*P*)	Effect size	Estimate (SE) *P*
BBSP_KGI	δ^13^C	1.90 (*P* = 0.372)	0.143	
(*n* = 39)	δ^15^N	1.74 (*P* = 0.028)	0.237	
	breeding stage		0.150	0.27(0.34) *P* = 0.427
WISP_KGI	δ^13^C	3.64 (*P* = 0.009)	0.568	
(*N* = 49)	δ^15^N	6.73 (*P* < 0.001)	0.719	
	breeding stage		0.552	0.39(0.25) *P* = 0.136
WISP_KER	δ^13^C	6.62 (*P* = 0.003)	0.857	
(*N* = 31)	δ^15^N	7.55 (*P* < 0.001)	0.908	
	breeding stage		0.882	0.10(0.12) *P* = 0.444

*Species*: *BBSP* Black-bellied Storm-petrel, *WISP* Wilson’s Storm-petrel, Sites: *KGI* King George Island/25 de Mayo Island (South Shetlands, Antarctic), *KER* Kerguelen Islands (Southern Indian Ocean)

Similarly, in models run across species, the trophic position according to CSIA-AA had a positive correlation with blood Hg values, while carbon stable isotope values did not correlate with Hg concentrations (Fig. S1). Overall, however, the strongest effects were those of species and site differences (Fig. S1).

The trophic position according to CSIA-AA measured in blood ranged from 2.9 to 4.0 (Table 3, Fig. S2), while the ranges for species overlapped, a 0.3–0.4 TP difference was seen within the Antarctic storm-petrels, with higher trophic levels in BBSP than WISP (Table 3, Fig. S2).

## Discussion

This study provides for the first time a large-scale assessment of Hg contamination in storm-petrels. While some populations (GBPS; WISP, BBSP from Kerguelen) showed values that are below toxicological risk thresholds, the BBSP from King George Island as well as the three northern hemisphere species (ASSP, BLSP, LESP) had feather Hg values above 5 µg/g dry weight, indicating a moderate toxicological risk (Burger and Gochfeld 1997).

### Spatial Differences in Hg

We first tested whether the level of contamination increases with a species’ more northerly breeding and inter-breeding distribution as Antarctic waters seem to be less contaminated than more northern waters (evidence from several seabird species: Cherel et al. 2018; Carravieri et al. 2016, 2017, 2020). This hypothesis was largely supported, but a notable exception was BBSP that had higher feather and blood Hg concentrations than other Antarctic species. Their Hg levels were comparable to those of storm-petrels breeding in the Mexican Pacific Islands. To the best of our knowledge, few storm-petrel populations have been studied for Hg so far. In the northern hemisphere, studies focussed only on the Atlantic sector (Table S1). The present data from three storm-petrel species from the Eastern Pacific Ocean show a comparable level of Hg contamination to species in the Northern Atlantic (Pollet et al. 2022, Table S1a).

In feathers, elevated Hg concentrations were only observed in the BBSP population from King George Island/25 de Mayo Island (Antarctica), suggesting that they accumulate Hg during their yearly cycle, which includes winter months in tropical waters of the Atlantic or Pacific Ocean. In comparison, BBSP from the Kerguelen Islands had lower Hg values in feathers, suggesting that they had not accumulated such high Hg stores throughout their annual migration, which is probably restricted to the Indian Ocean. A comparison of Hg concentrations of individual feathers (subsamples of *N* = 6 per colony) suggested that feathers sampled from individual BBSP were relatively similar to each other (repeatability 81%) in the King George Island/25 de Mayo Island population.

In contrast, we observed high intra-individual variability in most of the storm-petrels studied here. This may be an indication of moult strategies and yearly movements. Storm-petrels have different moult strategies in relation to their breeding and wintering sites. As soon as Antarctic species such as the WISP finish breeding, they fly to their wintering areas where they moult rapidly (Scott 1970). In contrast, temperate species may begin moult late during nesting and require the entire non-breeding period to complete their moult (Scott 1970). Thus, in those temperate species higher variability in Hg in their plumage is expected. In addition, differences in their year-round ecology and movements may also determine the moult schedule. For example, Ainley et al. (1976) compared the moult of ASSP and LESP at South Farallon, California. ASSP is a short-ranging, sedentary species, while LESP is a long-ranging, migratory species. The moult began for both species with a renewal of body feathers. LESP started body moult in the fall and finished in the spring, taking a mean of 274 days. ASSP needed less time (257 days), and had a different timing, starting moult at the time their eggs had hatched, and showing peak scores in August when adults were feeding their chicks (Ainley et al. 1976). In our analysis, the highest repeatability values of intra-individual feather Hg concentrations were found in the two migratory Antarctic populations (WISP: 52%, BBSP: 81%), and the sedentary population (ASSP: 58%), while lowest repeatability values were found in migratory northern hemisphere populations (BLSP 0%, LESP: 18%). This suggests that the latter two populations have extended moult durations over which they use waters of different Hg contamination degree or different prey, including breeding site, migration and winter areas. In albatross, prolonged moulting has been shown to affect the feather Hg concentrations dramatically (Cherel et al. 2018). In contrast, Antarctic storm-petrels moult after arrival to their winter site and at a faster rate (e.g. 4 months or 120 days in WISP, Stresemann and Stresemann 1966), leading to less intra-individual variability.

### Species Differences and Trophic Position

We further aimed to test if the level of contamination increases with the trophic position. We found some support for this hypothesis, but also some discordance in the results.

Among species, a linear model for feathers and blood supported a positive correlation between the trophic position according to CSIA-AA and Hg concentrations (Fig. S1). However, the species and site effects were much more important than those of the trophic position. This may be explained by the relatively similar trophic positions among storm-petrels, but also by the differences in time integration: while stable isotopes in feathers integrate nutrients taken up during the moulting time, Hg concentrations reflect the cumulative annual burden.

**Fig. 7:**
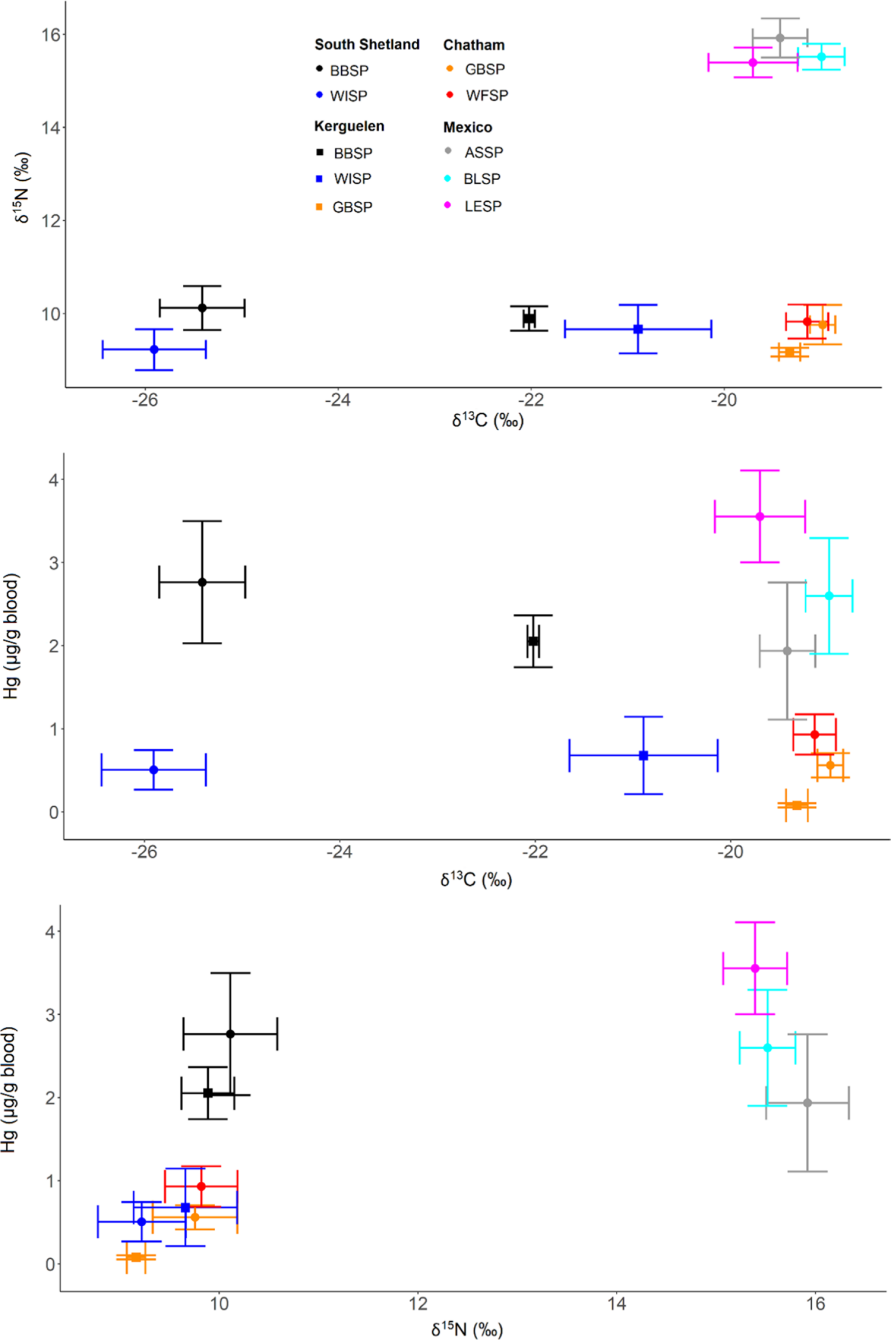
Hg concentrations and stable isotope values in blood of storm-petrels from ten populations, sampled during the chick-provisioning period. Species: ASSP = Ashy Storm-petrel, BBSP = Black-bellied Storm-petrel, BLSP = Black Storm-petrel, GBSP = Grey-backed Storm-petrel, LESP = Leach’s Storm-petrel, WFSP = White-faced Storm-petrel, WISP = Wilson’s Storm-petrel

In northern hemisphere habitats such as the California current, higher baseline δ^15^N values (e.g. Bedolla-Guzmán et al. 2021) were observed in seabirds, in line with our measurements (e.g. Figure 7). In a study comparing BLSP and LESP using stable isotopes, both species preyed on fish larvae in similar proportions (about 50%), but BLSP consumed higher trophic level krill (including *Nyctiphanes simplex*, *Nematoscelis difficilis*, and *Thysanoessa spinifera*), resulting in higher δ^15^N values (e.g. Bedolla-Guzmán et al. 2021). In our study, ASSP had the highest δ^15^N values in feathers and blood of the three species in the Mexican Pacific, but had similar Hg values to the other two species. These observations suggest that although the trophic position is an important factor influencing Hg contamination, other species-specific factors may also play a role, such as the type of prey. For example, myctophids can play an important role in the diet of storm-petrels (e.g. Croxall and North 1988; Vermeer and Devito 1988). Thus, all otoliths found in BBSP at King George Island/25 de Mayo Island were from the myctophid *Electrona antarctica* (Hahn 1998), and myctophids were key components of LESP in Atlantic Canada (Frith et al. 2020). Myctophids have notably high Hg concentrations (e.g. Seco et al. 2020: myctophid *Electrona antarctica*: 0.18 ± 0.09 μg g^−1^) compared to krill (Seco et al. 2019: *Euphausia tricantha*: 0.03 ± 0.01 μg g^−1^) and other higher trophic level prey such as predatory amphipods (Seco et al. 2021: *Themisto gaudichaudii*: 0.04 ± 0.01 μg g^−1^). Similar elevated Hg concentrations in myctophids compared to zooplankton species were reported in subantarctic Kerguelen waters (Bustamante et al. 2003; Cipro et al. 2018). Myctophids are mesopelagic and spend the day at the oxygen minimum zone where anaerobic microorganisms methylate inorganic Hg into MeHg (Martin et al. 2006). Consequently, storm-petrels that feed on myctophid fish coming to the surface at night may have high Hg concentrations in their tissues (Elliott and Elliott 2016). Feeding on mesopelagic prey explains high Hg concentrations in seabirds (Ochoa-Acuña et al. 2002) and thus, high Hg concentrations in BBSP from this study might result from feeding on myctophids, and species differences among the sympatric species in the Mexican Pacific.

### Seasonal Differences in Hg

By comparing blood samples collected early and late in the breeding season of the two Antarctic storm-petrel species, we aimed to test the hypothesis that the level of contamination is higher at the beginning of the season, as a result of carry-over from the winter areas. Over the course of the season, the contamination level should drop as birds spend more time in Antarctic waters where they feed on prey with lower Hg concentrations compare to the prey from lower latitudes. Hg decreases in blood over the course of the breeding season have been shown in seabirds which migrate to more contaminated areas in the non-breeding season (e.g. Double-Crested Cormorants *Phalacrocorax auratus*: Lavoie et al. 2014, Blue Petrels *Halobaena caerulea*: Quillfeldt et al. 2022a, Black-legged Kittiwakes: Tartu et al. 2022; Great skua *Stercorarius skua*: Albert et al. 2022). In the present study, a decrease was seen for WISP on King George Island/25 de Mayo Island (Antarctica), but not for WISP on the Kerguelen Islands (Southern Indian Ocean) or BBSP on King George Island/25 de Mayo Island. This suggests that for these latter populations, Hg exposure is similar in the breeding and non-breeding season. Colony-specific distributions from tracking data would be needed to explain these patterns.

## Conclusion

In summary, Hg contamination varied considerably among storm-petrels with large differences among species and sites. Even Antarctic storm-petrels that are far removed from sources of pollution, can experience considerable Hg contamination. The lowest contamination was observed in subantarctic species and populations. Further research is needed on the distribution and diet of these species, as well as the Hg contamination of their prey, in order to fully understand the observed patterns.

## Supplementary Information

Below is the link to the electronic supplementary material.

Supplementary file1 (PDF 532 KB)

## Data Availability

All raw data will be submitted to the PANGAEA database. Until publication in the database, raw data are available from the authors.
