# Supplementary material for: Mercury in Ten Storm-Petrel Populations from the Antarctic to the Subtropics
Source: Arch Environ Contam Toxicol. 2023 Jul 13;85(1):55–72. doi: 10.1007/s00244-023-01011-3 (PMC10374726; doi:10.1007/s00244-023-01011-3)
Supplement: Supplementary file 1 — Supplementary file1 (PDF 532 KB) [file 244_2023_1011_MOESM1_ESM.pdf]

Supplementary Information

Mercury in ten storm-petrel populations from the Antarctic to the Subtropics

Authors: Petra Quillfeldt, Yuliana Bedolla-Guzmán, Marcela M. Libertelli, Yves Cherel, Melanie Massaro & Paco Bustamante

Fig. S1. Linear model outputs of Hg in feathers (a,b) and blood (c,d) of storm-petrels from ten populations, in relation to $\delta^{13}C$, and the trophic position calculated from CSIA-AA.

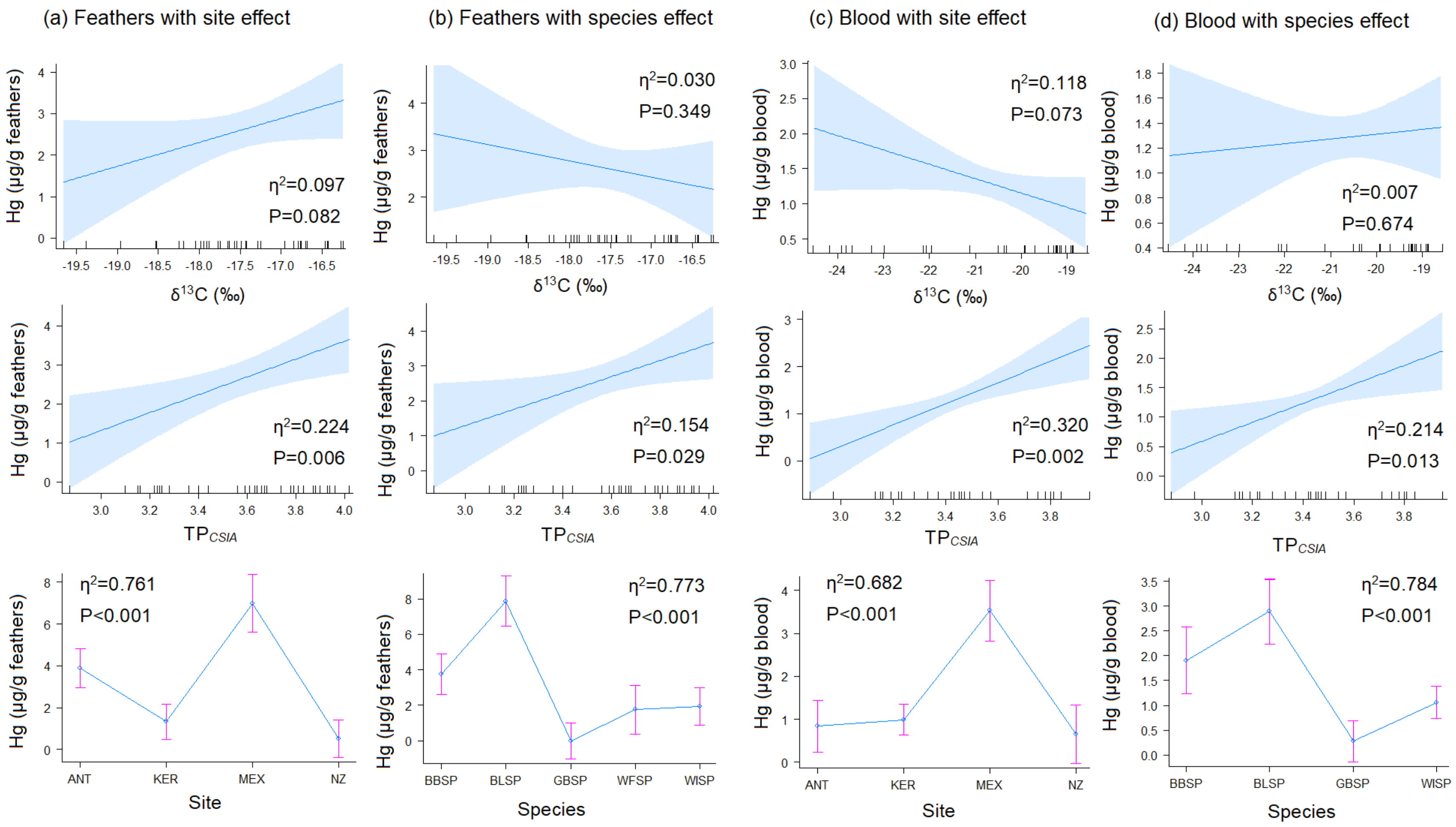

Fig. S2. Trophic positions according to CSIA-AA (Compound-specific stable isotope analysis of amino acids) in (a) feathers and (b) blood of storm-petrel populations. Species: BBSP=Black-bellied Storm-petrel, BLSP=Black Storm-petrel, GBSP=Grey-backed Storm-petrel, WFSP=White-faced Storm-petrel, WISP=Wilson's Storm-petrel, Sites: KGI=King George Island (South Shetlands, Antarctica), KER=Kerguelen (Southern Indian Ocean), MEX=Mexican Pacific Islands, CHA=Chatham Islands, New Zealand.

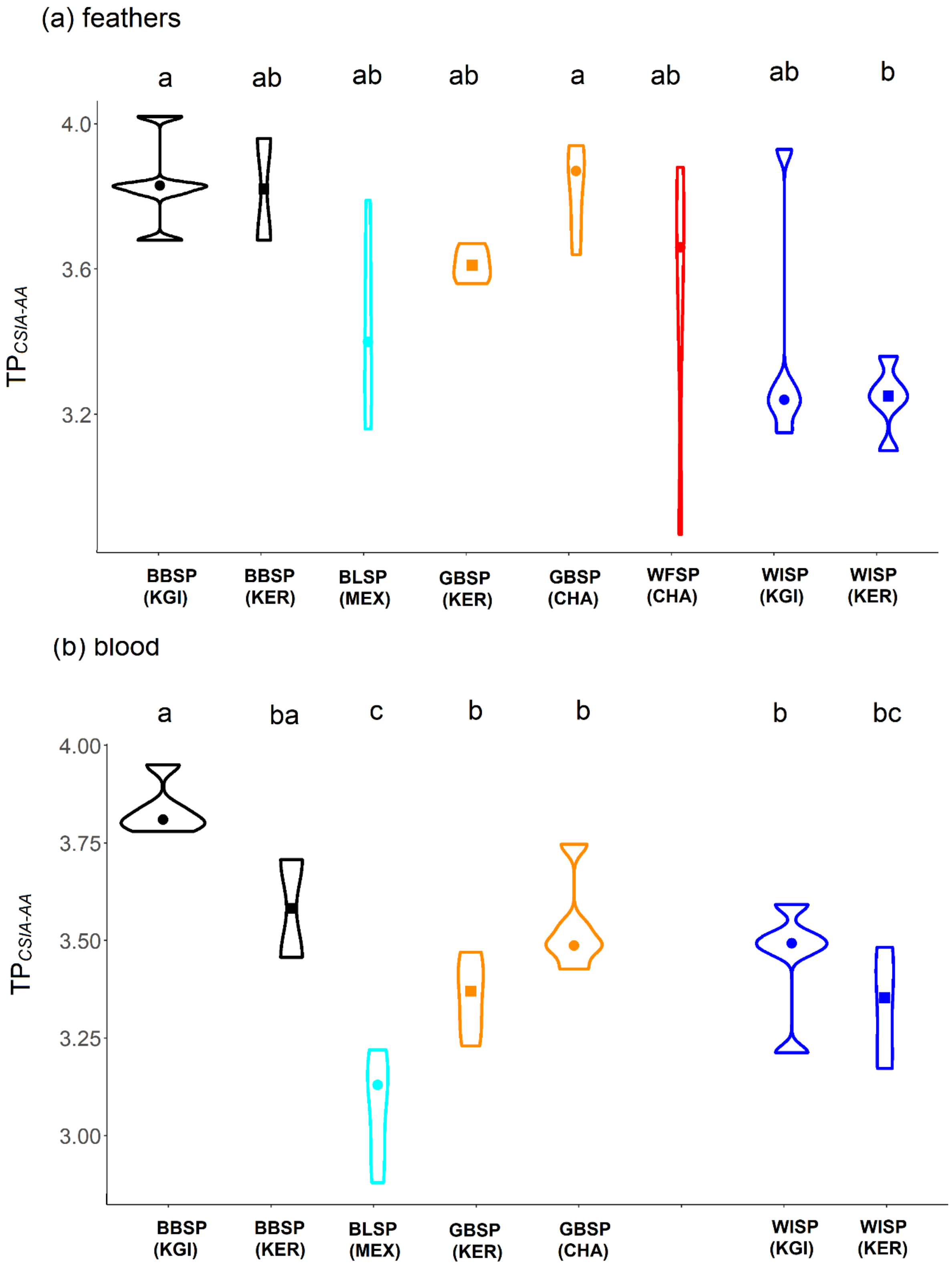

Fig. S3. Examples of Hg concentrations and stable isotope values in individually measured feathers, in a species with unrepeatable Hg values (Leach's Storm-petrel LESP from Mexican Pacific Islands) and two species with moderately repeatable Hg values (Ashy Storm-petrel from Mexican Pacific Islands and Black-bellied Storm-petrel King George Island/25 de Mayo Island (South Shetlands, Antarctica). A total of 4 body feathers per bird were measured.

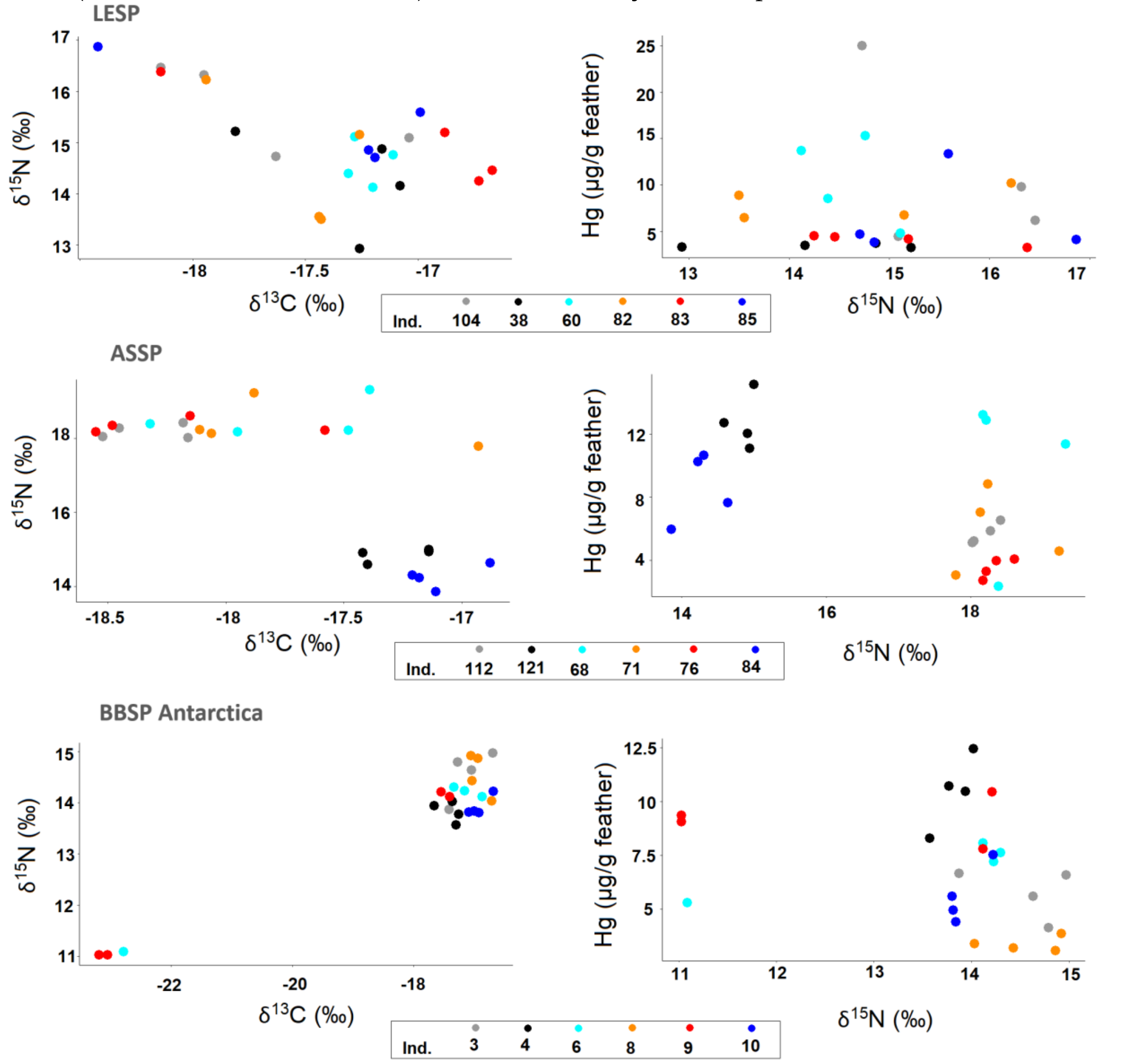

Fig. S4. General additive model (gam) fits of Hg values in feathers of storm-petrels breeding on King George Islands/25 de Mayo Island (South Shetland, Antarctic) and Kerguelen Islands, in relation to carbon and nitrogen stable isotope values and the time in the breeding season. Species: BBSP=Black-bellied Storm-petrel, WISP=Wilson’s Storm-petrel, Sites: KGI=King George Island/25 de Mayo Island (South Shetlands, Antarctic), KER=Kerguelen (Southern Indian Ocean)

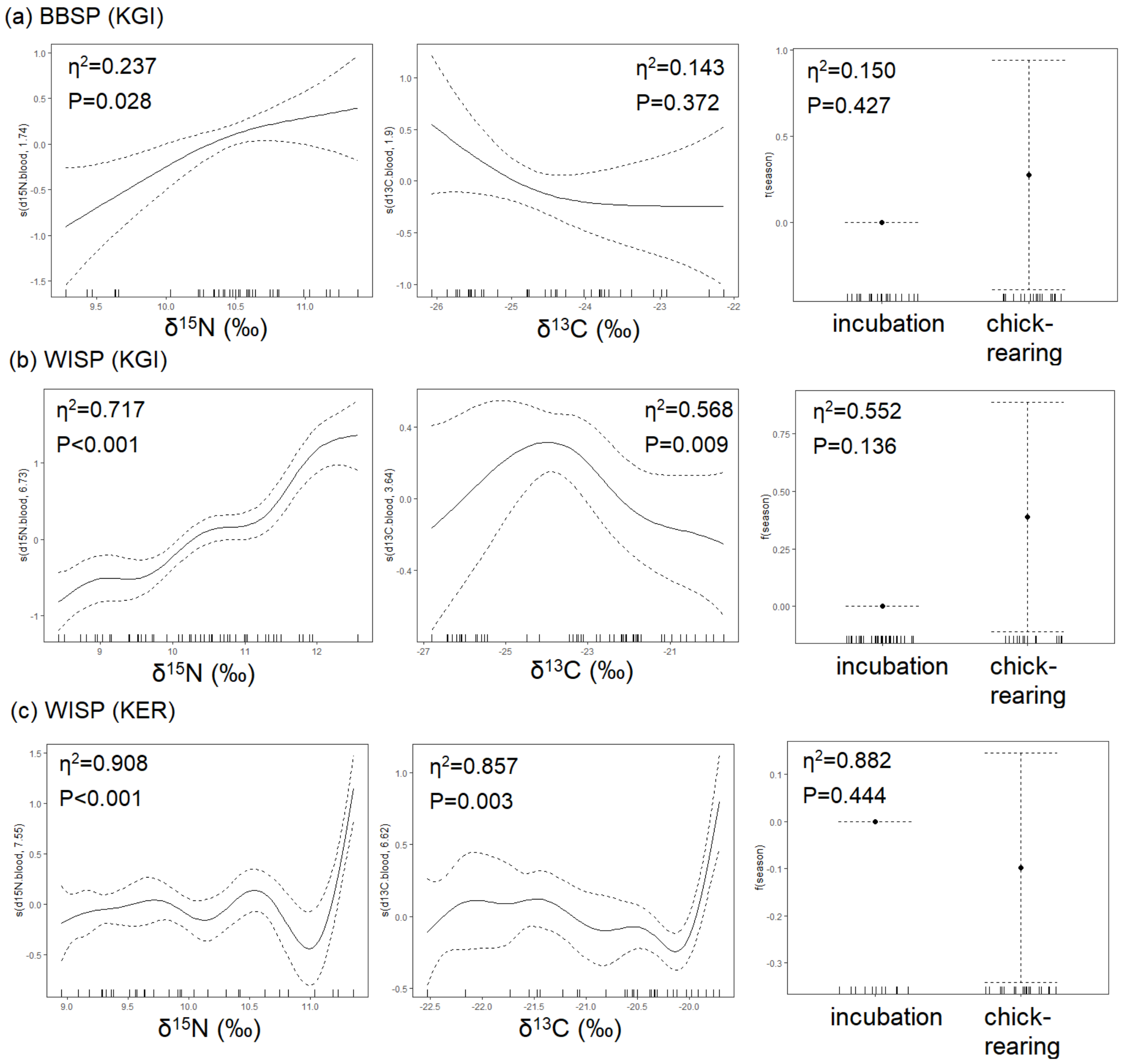

Table S1a. Previous data on Hg in feather and blood samples in Hydrobatidae. Data available in the literature for feather samples (body feathers).

Population		Sample type			Hg (µg.g^{-1} dry weight)	Reference
Storm-petrel	Colony site	age	tissue	Collection date	mean ± SD (n)	
H. furcata	Aleutians, Alaska	adults	feathers	2005-2006	6.70 ± 1.64 (12)	Kaler et al. 2014
H. castro	Azores	adults	feathers	1993-1995	17.4 ± 0.4 (130)	Monteiro et al. 1998
	Berlengas	adults	feathers	1993-1995	14.3 ± 3.7 (31)	Monteiro et al. 1999
	Madeira	adults	feathers	1993-1995	13.7 ± 2.6 (15)	Monteiro et al. 1999
	Salvages	adults	feathers	1993-1995	15.6 ± 3.8 (17)	Monteiro et al. 1999
H. monteroi	Azores	adults	feathers	1993-1995	11.1 ± 0.3 (100)	Monteiro et al. 1998
H. leucorhoa	New Brunswick, Canada	adults	feathers	2005-2006	4.80 ± 0.62 (5)	Bond & Diamond 2008
	Bay of Fundy, Canada	adults	feathers	2006	4.85 ± 2.79 (5)	Bond & Diamond 2009a
	Bay of Fundy, Canada	adults	feathers	2005-2006	7.00 (95% CI: 2.88-12.65)(15)	Bond & Diamond 2009b
	Bay of Fundy, Canada	chicks	feathers	2005-2006	1.42 (95% CI: 0.90-2.22)(20)	Bond & Diamond 2009b
	Bon Portage Island, Canada	adults	blood	2011-2015	3.91 ± 2.14 (90)	Pollet et al. 2017
	Northwest Atlantic (9 colonies)	adults	blood	2013-2019	3.62 ± 7.71 (742)	Pollet et al. 2022

Table S1b. Previous data on Hg in feather and blood samples in Oceanitidae. Data available in the literature for feather samples (body feathers and chick down).

Population		Sample type			Hg (µg.g^{-1} dry weight);	Reference
Storm-petrel	Colony site	age	tissue	Collection date	mean ± SD (n)	
O. leucorhoa	Machias Seal Island	adults	feathers	2005-2006	0.122 (16)	Bond et al. 2009
O. leucorhoa	Machias Seal Island	chicks	feathers	2005-2006	0.023 (19)	Bond et al. 2009
O. leucorhoa						Goodale et al. 2008
O. oceanicus	Kerguelen Islands	adults	feathers	2005	0.42 ± 0.13 (12)	Carravieri et al. 2014
	Chilean cost	adults	feathers	1995	1.7 ± 0.6 (2 ♀)	Ochoa-Acuña et al. 2002
	Chilean cost	adults	feathers	1995	1.8 ± 0.5 (7 ♂)	Ochoa-Acuña et al. 2002
	South Shetlands	adults	feathers	2008-09	2.88 ± 0.8 (8)	Becker et al. 2019
	South Shetlands	adults	feathers	2017	2.38 ± 1.47 (25)	Pacyna et al. 2019
	South Shetlands	chicks	feathers	2017	0.67 ± 0.27 (5)	Pacyna et al. 2019
	South Shetlands	chicks	down	2017	1.72 ± 0.65 (16)	Pacyna et al. 2019
F. tropica	Kerguelen Islands	adults	feathers	2005, 2010	4.22 ± 2.53 (10)	Carravieri et al. 2014
	South Shetlands	adults	feathers	2017	5.47 ± 1.61 (8)	Pacyna et al. 2019
	South Shetlands	chicks	feathers	2017	1.87 ± 0.29 (5)	Pacyna et al. 2019
	South Shetlands	chicks	down	2017	3.99 ± 1.07 (6)	Pacyna et al. 2019
F. grallaria	Chilean cost	adults	feathers	1995	3.8 (1)	Ochoa-Acuña et al. 2002
G. nereis	Kerguelen Islands	adults	feathers	2006	0.51 ± 0.44 (23)	Carravieri et al. 2014
	Gough Island	adults	feathers	2009	1.98 ± 2.07 (2)	Becker et al. 2019
P. marina	Gough Island	adults	feathers	2009	1.41 ± 0.44 (10)	Becker et al. 2019

References for Tables S1a and S1b:

Becker, P. H., Goutner, V., Ryan, P. G., & González-Solís, J. (2016). Feather Hg concentrations in Southern Ocean seabirds: variation by species, site and time. *Environmental Pollution, 216*, 253-263.

Bond, A. L., & Diamond, A. W. (2008). High within-individual variation in total Hg concentration in seabird feathers. *Environmental Toxicology and Chemistry: An International Journal*, *27*, 2375-2377.

Bond, A. L., & Diamond, A. W. (2009a). Total and methyl Hg concentrations in seabird feathers and eggs. *Archives of Environmental Contamination and Toxicology*, *56*, 286-291.

Bond, A. L., & Diamond, A. W. (2009b). Hg concentrations in seabird tissues from machias Seal Island, New Brunswick, Canada. *Science of the Total Environment*, *407*, 4340-4347.

Carravieri, A., Cherel, Y., Blévin, P., Brault-Favrou, M., Chastel, O., & Bustamante, P. (2014). Hg exposure in a large subantarctic avian community. *Environmental Pollution*, *190,* 51-57.

Kaler, R. S., Kenney, L. A., Bond, A. L., & Eagles-Smith, C. A. (2014). Hg concentrations in breast feathers of three upper trophic level marine predators from the western Aleutian Islands, Alaska. *Marine Pollution Bulletin, 82,* 189-193.

Monteiro, L. R., Granadeiro, J. P., & Furness, R. W. (1998). Relationship between Hg levels and diet in Azores seabirds. *Marine Ecology Progress Series, 166,* 259-265.

Monteiro, L. R., Granadeiro, J. P., Furness, R. W., Oliveira, P. (1999). Contemporary patterns of mercury contamination in the Portuguese Atlantic inferred from mercury concentrations in seabird tissues. Mar. Environ. Res. 47, 137–156.

Ochoa-Acuna, H., Sepúlveda, M.S., Gross, T.S. (2002) Mercury in feathers from Chilean birds: influence of location, feeding strategy, and taxonomic affiliation. *Marine Pollution Bulletin*, 44, 340-5.

Pacyna, A. D., Jakubas, D., Ausems, A. N., Frankowski, M., Polkowska, Ż., & Wojczulanis-Jakubas, K. (2019). Storm petrels as indicators of pelagic seabird exposure to chemical elements in the Antarctic marine ecosystem. *Science of the Total Environment*, *692*, 382-392.

Pollet, I. L., Leonard, M. L., O'Driscoll, N. J., Burgess, N. M., Shutler, D. (2017). Relationships between blood mercury levels, reproduction, and return rate in a small seabird. *Ecotoxicology*, 26, 97-103.

Pollet, I. L., McFarlane-Tranquilla, L., Burgess, N. M., Diamond, A. W., Gjerdrum, C., Hedd, A., Mallory, M. L. (2022). Factors influencing mercury levels in Leach's storm-petrels at northwest Atlantic colonies. *Science of The Total Environment,* 160464
